# Early Results of Mechanochemical Ablation by Flebogrif and Radiofrequency Ablation in Treating Primary Varicose Veins of Lower Limb

**DOI:** 10.7759/cureus.45874

**Published:** 2023-09-24

**Authors:** Abdul Razaque, Muhammad K Shazlee, Saqib Qamar, Syed Muhammad Shahnawaz Hyder, Hatem Adel

**Affiliations:** 1 Radiology, Indus Hospital and Health Network, Karachi, PAK; 2 Radiology, The Indus Hospital, Karachi, PAK; 3 Interventional Radiologist, Indus Hospital and Health Network, Karachi, PAK

**Keywords:** varicose veins, flebogrif, moca, rfa, chronic venous insufficiency

## Abstract

Introduction

Chronic venous insufficiency is a common condition that leads to the development of incompetent great or short saphenous veins (GSV or SSV) resulting in varicose vein development. Conservative management is initially employed for its treatment; however, the varicosities that do not respond to conservative management may require intervention by surgery or endovenous routes. Radiofrequency ablation (RFA) and mechanochemical ablation (MOCA) are the two most common endovascular techniques available for the management of incompetent GSV or SSV. Clarivein and Flebogrif are two devices to treat incompetent GSV or SSV by MOCA. Mechanical ablation is provided by their flexible cutting elements and chemical ablation is provided by polidocanol or sodium tetradecyl sulfate (STS). RFA uses radiofrequency waves to treat venous insufficiency. Therefore, the aim of this study was to determine the early treatment outcome results for incompetent GSV or SSV treated with RFA or MOCA by Flebogrif.

Materials and methods

This was a retrospective cross-sectional study undertaken at the Radiology Department of Indus Hospital and Health Network. Electronic Medical Records of all the patients who underwent RFA or MOCA for GSV or SSV for venous insufficiency from January 2021 to December 2021 were included. Both male and female patients aged 18 years and above diagnosed with venous insufficiency having Clinical, Etiologic, Anatomic, and Pathophysiologic (CEAP) scores of >1 were included. Statistical Package for Social Sciences (SPSS) v 22 (IBM Corp., Armonk, NY) was used for data entry and analysis.

Results

137 patients were included in the present study with a mean age of 53.8 ± 12.1 years. Pre-procedure CEAP score was C3 in 59 (84.4%), C4 in four (5.7%), and C6 in seven (10.0%) patients in patients who underwent RFA, and it was successful in 69 (98.6%) patients. Pre-procedure CEAP score was C3 in 62 (92.5%), C4 in two (3.0%), and C6 in three (4.5%) patients who underwent MOCA, and it was successful in 59 (88.1%) patients. Pain was the most frequent complication observed in both RFA and MOCA.

Conclusion

RFA has a high success rate as compared to MOCA by Flebogrif in treating incompetent GSV or SSVs.

## Introduction

Chronic venous insufficiency is a disease of venous reflux and is a common condition that serves as an underlying cause of varicose veins, reticular veins, or venous telangiectasia. Endovascular methods can readily treat superficial venous reflux [1-3]. Usually, a failure of venous valves of the great saphenous vein (GSV) or short saphenous veins (SSV) leads to venous reflux. The symptoms of reflux include dilated veins which are visible on the skin, lower extremity swelling, discoloration of the skin, lower extremity heaviness, and propensity to ulcer formation [4,5].

Chronic venous insufficiency has a prevalence of approximately 34.8% in Pakistan [6]. This condition is conservatively managed by long-term use of compression stockings, leg elevation, and oral pain medications [7]. Those conditions not controlled with conservative management require intervention. These interventional methods include stripping or ligation of incompetent veins or endovascular management by endovenous laser ablation, radiofrequency ablation (RFA), or mechanochemical ablation (MOCA) [8-10]. Clarivein and Flebogrif are two devices to treat incompetent GSV or SSV by MOCA. Mechanical ablation is provided by their flexible cutting elements and chemical ablation is provided by polidocanol or sodium tetradecyl sulfate (STS). RFA uses radiofrequency waves to treat venous insufficiency. A previous study showed completely successful outcomes of 98.2% by RFA [11] and 87% by MOCA [9]. Another study shows a high success rate of MOCA [12].

Venous insufficiency has a significant impact on quality of life. Therefore, prompt treatment should be carried out to prevent the development of complications such as venous ulcers. To the best of our knowledge and after going through various search engines such as Pubmed, Google Scholar, and Scopus, the outcome of RFA and MOCA for treatment of ablation of GSV or SSV have not been compared. Therefore, this study will determine the early outcome results at 6 months for incompetent GSV or SSV treated with RFA or MOCA by Flebogrif, which is a new minimally invasive technique to treat varicose veins. The mechanical damage is done by cutting hooks of the Flebogrif and chemical damage is done by sclerosing agents. Clarivein is another instrument used for MOCA but is not available in our country.

## Materials and methods

**Figure 1:**
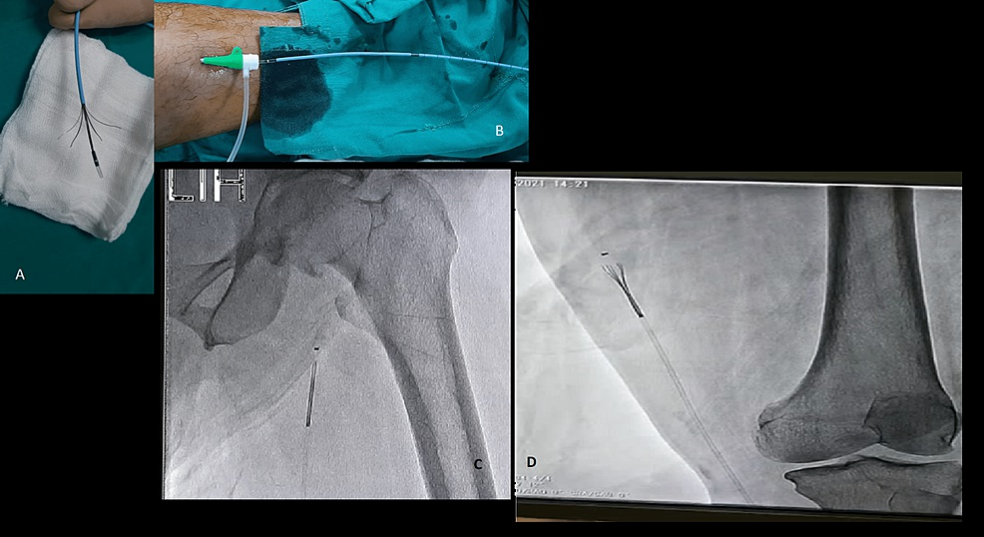
Flebogrif system. (A) Flebogrif with its fangs open. (B) Flebogrif placed in the sheath. (C) Placement of Flebogrif in GSV. (D) Flebogrif open within GSV.

This was a retrospective cross-sectional study undertaken at Radiology Department of Indus Hospital and Health Network, Karachi, Pakistan. Electronic medical records (EMR) of all the patients who underwent RFA or MOCA for GSV or SSV for venous insufficiency from January 2021 to December 2021 were included. The requirement for institutional approval was waived as all the data was retrieved from the EMR of the system. The criteria for inclusion included both male and female patients aged 18 years and above and diagnosed with venous insufficiency having Clinical, Etiologic, Anatomic, and Pathophysiologic (CEAP) scores of >C1. Exclusion criteria of the study were patients with previous or current deep venous thrombosis, patients having severely tortuous GSV or SSV, having CEAP score of C0-C1, or pregnant or lactating females. For RFA or MOCA, using the Seldinger technique, a guide wire was passed and a 6F vascular sheath was placed in GSV or SSV. RFA was performed using a closure FAST catheter. A 5-6 cm segment of vein was ablated for 20 seconds each at a temperature of 120ºC. MOCA was performed using a Flebogrif catheter as shown in Figures 1A-1D.

One ampule of STS measured approximately 2 mL and was diluted with 4 mL air and 2 mL distilled water and approximately 8 mL solution was made. It was injected slowly along with retraction of Flebogrif catheter retraction. Statistical Package for Social Sciences (SPSS) version 22 (IBM Corp., Armonk, NY) was used for data entry and analysis. Normality of data will be determined by Shapiro-Wilk test. Quantitative variables such as age were mentioned as mean and standard deviation. Qualitative variables such as pre-procedure and post-procedure CEAP score, outcome (presence or absence of recanalization), post-procedure complications (endothermal heat-induced thrombosis (EHIT), thrombophlebitis, pain/tenderness, paresthesia and infection) were mentioned as frequency and percentage. Effect modifiers such as age, gender and post procedure complications were be stratified to see their effect on outcome variables. Post stratification, chi-square test and Fischer exact test was applied and p-value of ≤0.05 was taken as significant.

## Results

One hundred and thirty-seven patients were included in the present study with a mean age of 53.8 ± 12.1 years (range 33-80 years). Table 1 describes the baseline characteristics of the patients.

**Table 1:** Baseline characteristics of the patients

Characteristics		%
Age, years	53.8 ± 12.1	
Height, cm	164.8 ± 4.9	
Weight, kg	64.6 ± 8.7	
Body Mass Index	23.6 ± 2.9	
Males	68	49.6
Females	69	50.4

Out of 70 patients who underwent RFA, female predominance was higher and was found in 38 (54.3%) patients. Pre-procedure CEAP score was C3 in 59 (84.4%), C4 in four (5.7%), and C6 in seven (10.0%) patients. RFA was successful in 69 (98.6%) patients with only one patient (1.4%) presenting with recanalization at six weeks follow-up. Post-procedure CEAP score was C0 in 61 (87.1%), C2A in five (7.4%), C5 in three (4.3%), and C6 in one (1.4%) patient. Pain was the most frequent complication observed in seven (10.0%) patients. EHIT was observed in one (1.4%) patient. Table 2 summarizes the characteristics of patients who underwent RFA.

**Table 2:** Characteristics of patients who underwent RFA

Gender	n	%
Males	32	45.7
Females	38	54.3
CEAP score before procedure		
C3	59	84.4
C4	4	5.7
C6	7	10.0
CEAP score after procedure		
C0	61	87.1
C2A	5	7.4
C5	3	4.3
C6	1	1.4
Outcome		
Successful	69	98.6
Failure	1	1.4
Complications		
Present	8	11.4
Absent	62	88.6
Type of complications		
Pain	7	10.0
Endothermal Heat Induced Thrombosis	1	1.4

Out of 67 patients who underwent MOCA by flebogrif, male predominance was higher and was found in 36 (53.7%) patients. Pre-procedure CEAP score was C3 in 62 (92.5%), C4 in 2 (3.0%) and C6 in three (4.5%) patients. MOCA was successful in 59 (88.1%) patients and failed in eight (11.9%), who developed recanalization at six weeks follow up. Post-procedure CEAP score was C0 in 52 (77.6%) and C2A in 15 (22.4%) patients. Pain was the most frequent complication observed following MOCA that was present in 10 (14.9%) patients followed by thrombophlebitis in three (4.5%) patients. Table 3 summarizes characteristics of patients who underwent MOCA by Flebogrif.

**Table 3:** Characteristics of patients who underwent MOCA by Flebogrif

Gender	n	%
Males	31	46.3
Females	36	53.7
CEAP score before procedure		
C3	62	92.5
C4	2	3.0
C6	3	4.5
CEAP score after procedure		
C0	63	94.0
C2A	6	6.0
Outcome		
Successful	59	88.1
Failure	8	11.9
Complications		
Present	13	19.4
Absent	54	80.6
Type of complications		
Pain	10	14.9
Thrombophlebitis	3	4.5

In patients who underwent RFA, successful outcomes were higher in females (p-value 0.543) and in patients aged more than 45 years (p-value 0.729) (Table 4).

**Table 4:** Comparison of outcome by RFA with patient characteristics

	Outcome		Total	P-value
	Successful	Failure		
Female	37 (52.9%)	1 (1.4%)	38 (54.3%)	
Male	32 (45.7%)	0 (0.0%)	32 (45.7%)	0.543^*^
Age ≤45 years	19 (27.1%)	0 (0.0%)	19 (27.1%)	
Age >45 years	50 (71.4%)	1 (1.4%)	51 (72.9%)	0.729^*^
Complications Absent	61 (87.1%)	1 (1.4%)	62 (88.6%)	
Complications Present	8 (11.4%)	0 (0.0%)	8 (88.6%)	0.886^*^
^*Fisher’s exact test applied^				

In patients who underwent MOCA by Flebogrif, successful outcomes were higher in males (p-value 0.270) and in patients aged greater than 45 years (p-value 0.525) (Table 5).

**Table 5:** Comparison of outcome by Flebogrif with patient characteristics

	Outcome		Total	P-value
	Successful	Failure		
Female	29 (43.3%)	2 (3.0%)	31 (46.3%)	
Male	30 (44.8%)	6 (9.0%)	36 (53.7%)	0.270^*^
Age ≤45 years	19 (28.4%)	3 (4.5%)	22 (32.8%)	
Age >45 years	40 (59.7%)	5 (7.5%)	45 (67.2%)	0.525^*^
Complications Present	12 (17.9%)	1 (1.5%)	13 (19.4%)	
Complications Absent	47 (70.1%)	7 (10.4%)	54 (80.6%)	0.513^*^
^*Fisher’s exact test applied^				

## Discussion

In this retrospective study, we determined the early outcomes in patients with venous insufficiency having incompetent GSV or SSV being treated by RFA or MOCA by Flebogrif. Our study results showed that procedure failure in the form of the development of recanalization of GSV was present in 11.3% of the patients. However, an Italian study by Ammollo et al. reported recanalization in 14.3% of cases, which is higher compared to our study [12]. A difference in failure rates could be attributed to the difference in the chemical used for ablation. In our study, we utilized STS, whereas previous studies used Polidocanol for ablation [12]. Another study showed almost similar success rates of MOCA at early follow-up, but later follow-ups showed reduced success rates of MOCA. In that study, Clarivein device was used for MOCA [13].

In the present study, the success rate of RFA at early follow was 98.6%. Jin et al. showed an initial success rate of 97.3% which is slightly lower as compared to our study [14]. That study was conducted in the Korean population with a sample size of 117 [14]. Li et al. showed almost comparable success rates of RFA in GSV occlusion [15]. The study was conducted in Chinese and, in this study, the ablation was aided by grade II compression of the iliac vein [15]. RFA treats the incompetent GSV or SSV by high-temperature heat waves. This technique is less invasive as compared to stripping surgery. Jeon et al. showed a slightly lower success rate of endovenous ablation of GSV by RFA [16]. Tamura et al. showed higher success rates of RFA as compared to our study. Our study reported a 98.6% success rate, but that study showed a 99% success rate [17].

It was reported in our study that the success rate of RFA was higher as compared to MOCA. However, Lane et al. reported that the occlusion rates of RFA and MOCA were similar [18]. This study was conducted on 170 patients and the follow-up duration in that study was 21 months as compared to six months in our study. Holewijn et al. also showed similar clinical outcomes of RFA and MOCA for GSV incompetence [19]. However, their study design was a randomized controlled trial and the sample size was 213 which was larger compared to our study. Another clinical trial reported almost similar results [20]. Vähäaho et al. in their study conducted on 117 patients showed almost similar results to our study, indicating that MOCA has a relatively lower success rate as compared to RFA [21].

Among complications caused by MOCA and RFA, pain was the most common complication observed in our study. It was labeled qualitatively as reported by the patient by taking history from them. Pain was higher in patients who underwent MOCA as compared to patients who underwent RFA. These results contrast with other international trials, which report that post-operative pain was lower in patients with MOCA as compared to patients with RFA [19,20]. In patients who underwent RFA, EHIT was reported in one patient. In that patient, focal thrombosis was noted in the deep vein. Thrombophlebitis was observed in three patients in our study who underwent MOCA. A reason for increased pain could be hypothesized to difference in ethnicity and also a difference in tolerating pain threshold.

However, our study is not without certain limitations. Our study was a retrospective single-center study. A second limitation of this study was the small sample size. The third limitation of this study was that the effect of the presence of incompetent perforators was not recorded. The fourth limitation of our study was that MOCA was performed using STS. Internationally, MOCA is usually performed using different strengths of polidocanol [22]. Whereas STS can be prepared easily diluted with water for injection and air. The fifth limitation of our study was that in our study, long-term follow-up was not evaluated. Many studies have evaluated the clinical effectiveness of these treatments on long-term follow-ups. Despite these limitations, we believe that this study is a major step in the evaluation of MOCA and RFA in treating venous incompetence from a developing country perspective.

It is recommended that further prospective studies should be carried out on this topic incorporating variables such as the VAS score of pain, the effect of compression stockings post-procedure, and the use of pre-procedure sedation to see its effect on complications and outcomes.

## Conclusions

Treatment of GSV or SSV has been revolutionized by the introduction of minimally invasive techniques. Both techniques can be performed under local anesthesia. RFA and MOCA by Flebogrif are highly effective techniques for treating incompetent GSVs. In our study, the effectiveness of radiofrequency was higher as compared to Flebogrif.
